# Highly Efficient Ultracentrifugation-free Chromatographic Purification of Recombinant AAV Serotype 9

**DOI:** 10.1016/j.omtm.2018.10.015

**Published:** 2018-11-01

**Authors:** Taro Tomono, Yukihiko Hirai, Hironori Okada, Yoshitaka Miyagawa, Kumi Adachi, Shuhei Sakamoto, Yasuhiro Kawano, Hideto Chono, Junichi Mineno, Akiko Ishii, Takashi Shimada, Masafumi Onodera, Akira Tamaoka, Takashi Okada

**Affiliations:** 1 Department of Biochemistry and Molecular Biology, Nippon Medical School, Tokyo, Japan; 2 Department of Human Genetics, National Center for Child Health and Development, Tokyo, Japan; 3 CDM center, Takara Bio Inc., Shiga, Japan; 4 Department of Neurology, Faculty of Medicine, University of Tsukuba, Ibaraki, Japan; 5 Graduate School of Comprehensive Human Sciences, Majors in Medical Sciences, University of Tsukuba, Ibaraki, Japan

**Keywords:** adeno-associated virus, rAAV9, quaternary ammonium anion exchangers, ultracentrifugation-free protocol

## Abstract

Recombinant adeno-associated virus serotype 9 (rAAV9) can specifically transduce muscle and neuronal tissues; thus, rAAV9 can potentially be used in gene therapy. However, rAAV9 is the most challenging rAAV serotype to purify. Traditionally, rAAV9 has been purified by ultracentrifugation, which is not scalable. We recently described a chromatographic purification protocol for rAAV1; this protocol can achieve scalable purifications. In this study, we attempted to optimize this protocol for purifying rAAV9 preparations, and we developed a novel, effective method for high-yield purification of rAAV9 using quaternary ammonium anion exchangers and size-exclusion chromatography. The final purified rAAV9 contained mainly three capsid proteins, as observed by SDS-PAGE. Furthermore, negative-stain electron microscopy demonstrated that 96.1% ± 1.1% of rAAV9 particles carried the viral genome containing the EGFP transgene, indicating that impurities and empty capsids can be eliminated with our purification protocol. The final rAAV9 titer obtained by our protocol totaled 2.5 ± 0.4 × 10^15^ viral genomes produced from ∼3.2 × 10^9^ HEK293EB cells. We confirmed that our protocol can also be applied to purify other varied AAV genome constructs. Our protocol can scale up production of pure rAAV9, in compliance with current good manufacturing practice, for clinical applications in human gene therapy.

## Introduction

Recombinant adeno-associated viruses (rAAVs) have long been actively studied as gene delivery vectors. rAAVs generated using different natural AAV serotypes display different tissue tropisms *in vivo*; therefore, rAAVs have emerged as versatile delivery vehicles for gene therapy.^1^, ^2^, ^3^, ^4^ In fact, rAAVs have already shown potential in clinical trials and have been used for treating lipoprotein lipase deficiency and Leber congenital amaurosis.^5^, ^6^, ^7^ To extend the possibility of rAAV-based gene therapy in human, it is essential to improve the methodologies for rAAV production and purification. For clinical gene therapy applications, the final preparation of rAAV should contain as few impurities and empty capsids as possible to avoid inflammation and immune responses. Furthermore, purification protocols should preferably be expandable, cost-effective, and reproducible for mass production. Conventional rAAV purification methods use cesium chloride (CsCl) or iodixanol density gradient ultracentrifugation,^8^, ^9^ entailing cumbersome procedures, and are unsuitable for large-scale purification. Therefore, developing alternative rAAV preparation methods is necessary to satisfy these conditions.

Chromatographic purification is more suitable for large-scale vector purification than commonly used ultracentrifugation methods. Thus, several chromatographic protocols have been proposed for purifying rAAVs.^10^, ^11^, ^12^, ^13^, ^14^, ^15^, ^16^ Additionally, to exclude contaminating cellular proteins and genomic DNA while maintaining high biological activity, it is desirable to collect the secreted rAAV from culture media that exclude serum because minimal levels of cellular or serum contaminants can improve the loading capacity and the purification procedure.^17^ Fortunately, most AAV serotypes except AAV2 are secreted by transfected cells into the culture supernatant.^17^, ^18^ We have also demonstrated that rAAV1^10^, ^11^ and rAAV8^10^ are secreted by cells transfected with *cis*, *trans*, and helper AAV plasmids. Thus, collecting rAAVs secreted in the culture media, which exclude serum, and their chromatographic purification are applicable to optimal purification of most AAV serotypes, resulting in high purity and high yields.

Among the AAV serotypes, rAAV9 is expected to be used widely in human gene therapy because of its broad tissue tropism. For instance, rAAV9 can transduce the cardiac muscles more efficiently than serotype 8.^19^ In addition, it can cross the blood-brain barrier.^20^, ^21^, ^22^ Thus, rAAV9 is a potential therapeutic vector for cardiac, neurodegenerative, and neuromuscular diseases.^23^ Therefore, the production of high-quality rAAV9 particles will have a substantial effect on gene therapy. In this study, we have established a simple, ultracentrifugation-free protocol for the production of large quantities of highly pure rAAV9 preparations. In our protocol, the cell culture medium, which excludes serum, is applied to a quaternary ammonium anion exchanger, yielding highly pure rAAVs that can be verified by detecting its three major AAV capsid proteins by SDS-PAGE. An important modification is the use of the supernatant derived from HEK293 EB cells (HEK293 cells expressing the *E1* gene region [*E1A*, *E1B19K*, and *E2A*] and the *BCL-X*_*L*_ gene)^24^ transfected with the AAV *cis*, *trans*, and helper plasmids to produce high yields of rAAV9. Most of the contaminants and empty capsids in the culture supernatant can be separated from purified rAAV9 by this easy purification method. Thus, our simple protocol is capable of producing high-quality rAAV9 with a high manufacturing yield.

## Results

### Precipitation of rAAV9 by Ammonium Aulfate

**Figure 1:**
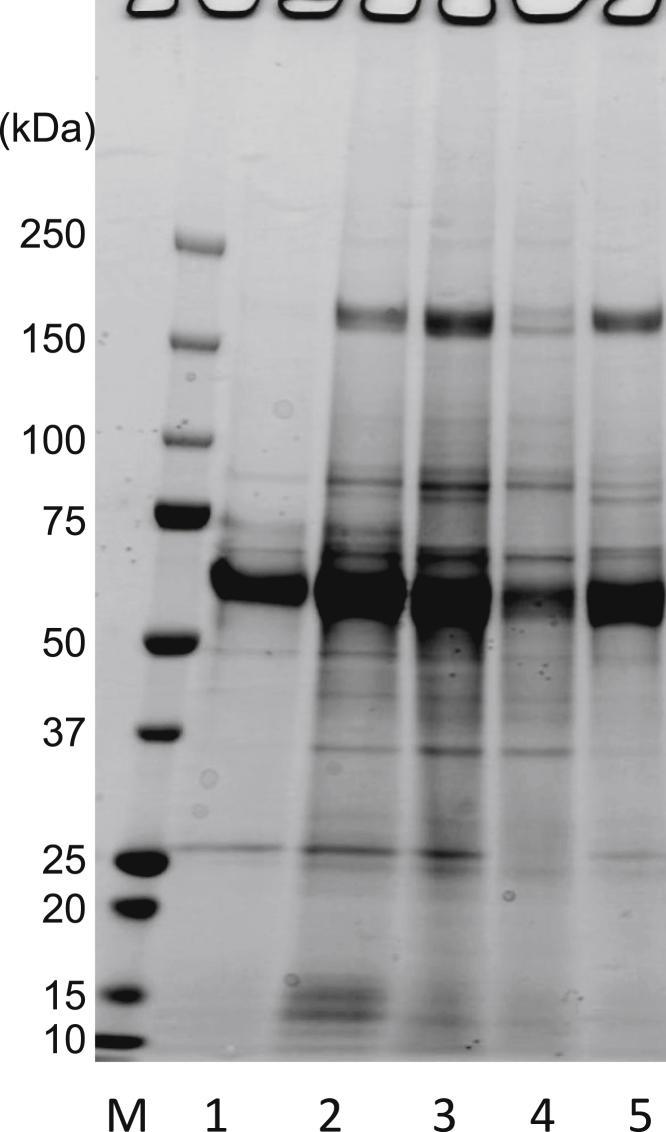
Initial Two-Step Treatment of Culture Supernatant with Saturated AS Precipitation AS was added to the post-tangential flow-filtration fraction (post-TFF) to achieve 1/3 saturation. AAV9-dsEGFP was finally precipitated in 1/2 AS solution (1/3→1/2 AS, lane 5). The post-TFF was treated with half-saturated AS (1/2 AS) precipitation alone as the conventional procedure (lane 3). Samples were analyzed by 5%–20% (v/v) gradient gel SDS-PAGE with Q-CBB staining. M, protein size marker; lane 1, pre-TFF; lane 2, post-TFF; lane 3, post-TFF preparation directly precipitated in 1/2 AS; lane 4, 1/3 AS precipitate; lane 5, 1/3→1/2 AS precipitate.

Previously, we successfully used ammonium sulfate (AS) to precipitate and concentrate rAAV1.^11^ Therefore, we first tested whether AS can be used to precipitate rAAV9. To test AS precipitation, rAAV9 (AAV9-double-stranded EGFP [dsEGFP], using pdsAAV-chicken β-actin [CBA]-EGFP) was produced by HEK293 cells (Materials and Methods). Figure 1 shows that a two-step precipitation procedure using sequential 1/3- and 1/2-saturated AS treatments (1/3→1/2 AS, lane 5) yielded better purity than a one-step procedure using only the 1/2-saturated AS treatment (1/2 AS, lane 3). Viral purity was determined by SDS-PAGE. The total protein amount following 1/3→1/2 AS treatment was 11% less than 1/2 AS treatment, as determined by densitometry of protein band intensities using ImageJ software. Many low-molecular-weight proteins were removed by 1/3→1/2 AS treatment (Figure 1, lanes 3–5). Thus, the AS treatment we describe is a viable method for rAAV9 precipitation.

### Purification of AAV9-dsEGFP by Quaternary Ammonium Anion Exchangers and Size-Exclusion Chromatography

**Figure 2:**
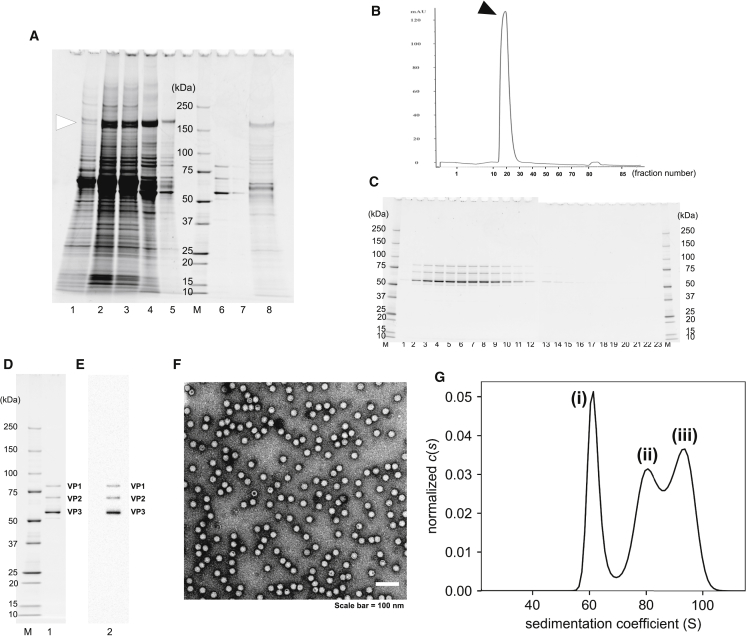
Laboratory-Scale Purification of AAV9-dsEGFP by Quaternary Ammonium Anion-Exchange Column and Size-Exclusion Chromatography (A) The AAV9-dsEGFP preparations were analyzed by 5%–20% (v/v) gradient gel SDS-PAGE and stained with Oriole fluorescent gel stain before and after chromatography purification using a HiPrep Q XL 16/10 column. The white arrowhead indicates a 200-kDa impurity. Lane 1, pre-TFF; lane 2, post-TFF; lane 3, after heat treatment; lane 4, 1/3→1/2 AS; lane 5, diluted 1/3→1/2 AS; lane 6, pass-through fraction; lane 7, wash-out fraction; lane 8, column-bound and eluted fraction. (B) The pass-through fraction was subsequently subjected to size-exclusion chromatography using a HiLoad 16/60 Superdex 200 preparation-grade column using an ÄKTA Explorer 100 HPLC system equipped with a 10-mL sample loop, with MHN buffer (pH 6.5) containing 300 mM NaCl and 0.01% (w/v) Pluronic F-68 as the mobile phase. y axis, 280 nm absorbance; x axis, fraction number. The black arrowhead indicates the peak fractions of the rAAV9 (corresponding to lanes 2–14 in C). (C) The elution fraction was analyzed by two 5%–20% (v/v) gradient SDS-PAGE gels with Oriole fluorescent staining; the left gel is from lanes 1–12, and the right gel is from lanes 13–23. Lanes 1–18, fractions 14–31; lane 19, fraction 33; lane 20, fraction 35; lane 21, fraction 37; lane 22, fraction 39; lane 23, fraction 41. Peak fractions (fractions 15–27) were collected to obtain the final product. (D–G) The final AAV9-dsEGFP product was analyzed by 5%–20% (v/v) gradient gel SDS-PAGE with Oriole fluorescent staining (D), western blotting (E, anti-AAV capsid monoclonal antibody B1), EM (F, negative staining), and analytical ultracentrifugation (AUC, G). Shown are the peak fractions of (i) empty particles (68.6 S, 25.7%), (ii) intermediate particles (88.8 S, 32.4%), and (iii) fully packaged virions (102.6 S, 38.8%). The goodness of fit (RMSD) was 0.004635. y axis, continuous-size C(S) distribution; x axis, sedimentation coefficient. Lane 1 and lane 2, the final rAAV9 product. The three bands represent the AAV9 capsid proteins VP1 (82 kDa), VP2 (67 kDa), and VP3 (60 kDa).

Next we used a chromatographic technique for laboratory-scale production of highly pure rAAV9. To increase the rAAV9 yield, we chose the HEK293EB cells, which express the *E1* gene region (*E1A*, *E1B19K*, and *E2A*) and the *BCL-X*_*L*_ gene; these cells yield 2-fold more rAAV than HEK293 cells.^24^ For the laboratory-scale purification, AAV9-dsEGFP was produced using 3.2 × 10^9^ HEK293EB cells (the volume of medium was 1,120 mL). After 1/3→1/2 AS treatment, the AAV9-dsEGFP sample was dissolved in 20 mL of 3.3 mM morpholinoethanesulfonic acid, 3.3 mM 4-(2-hydroxyethyl)-1-piperazineethanesulfonic acid, and 3.3 mM sodium acetate buffer (MHN buffer, dilution buffer; pH 8.0) containing 50 mM NaCl and 0.01% (w/v) Pluronic F-68. This purification method was based on the results of a preliminary small-scale experiment (Supplemental Materials and Methods; Figure S1). The 1/3→1/2 AS treatment was applicable to rAAV9 produced from HEK293EB cells. The 1/3→1/2 AS-treated crude AAV9-dsEGFP fraction was diluted in dilution buffer until the conductivity of the solution decreased to 7.3 mS/cm. A HiPrep Q XL 16/10 column with a bed volume of 20 mL was used for laboratory-scale purification. This column has the same specifications as the HiTrap Q FF column with a bed volume of 1 mL used for preliminary small-scale experiments. The diluted sample was loaded onto the HiPrep Q XL 16/10 column equilibrated with dilution buffer at a rate of 3 mL/min, achieved by a peristaltic pump P1. Figure 2A shows the three major protein bands present in the pass-through fraction (lane 6) and the protein impurities retained in the column-bound fraction (lane 8), consistent with the results of the preliminary small-scale experiment (using HiTrap Q FF; Figure S1). The 200-kDa impurity (white arrowhead in Figure 2A), which was difficult to remove during rAAV1 purification, was separated from the rAAV9 preparation just by loading onto the anion-exchange column. The pass-through fraction was concentrated using an Ultracel 30 K centrifugal filter unit. Finally, AAV9-dsEGFP was purified by size-exclusion chromatography (HiLoad 16/60 Superdex 200, preparation-grade) using an ÄKTA Explorer 100 high performance liquid chromatography (HPLC) system equipped with a 10-mL sample loop and MHN (pH 6.5) buffer containing 300 mM NaCl and 0.01% (w/v) Pluronic F-68. The peak indicated by a black arrowhead in the chromatogram (Figure 2B) and the protein bands in lanes 2–14 (Figure 2C) represent the rAAV9 particles. Peak fractions (fractions 15–27) were collected to obtain the final product. The resultant total titer of pure AAV9-dsEGFP was 2.9 × 10^15^ v.g. or 3.7 × 10^14^ vector genomes (v.g.), measured by qPCR using primers targeting the inverted terminal repeats (ITR) or EGFP, and the final product contained 3.8% (195 of 5,168 particles) of empty capsids, as determined by negative-stain electron microscopy (EM) (trial 1, Table 1). According to Figure S2, a certain level of empty capsids was observed in the diluted sample just before loading onto the anion-exchange column; thus, use of the anion-exchange column was enough to remove the empty particles. Taken together, our chromatographic procedure enables purification of high-quality rAAV9.

**Table 1:** Total Titer at Each Step (Pre-TFF, Post-TFF, Anion-Exchange Column Purification, and Final Product) and Recovery, Measured by qPCR, in Five Trials (Three Trials for AAV9-dsEGFP, One Trial for AAV9-dsLuc, and One Trial for AAV-ssLuc)

rAAV Genome	dsAAV-CBA-EGFP			dsAAV-CBA-RFLuc	ssAAV-CMV-RFLuc^a^
AAV genome form	ds			ds	ss
GC content	59%			55%	52%
Trial number	1	2	3	4	5
Total Titer					
Pre-TFF^b^^,^^c^	1.3 × 10^16^ v.g.	9.9 × 10^15^ v.g.	1.0 × 10^16^ v.g.	8.5 × 10^15^ v.g.	3.6 × 10^14^ v.g.
Post-TFF^c^	5.3 × 10^15^ v.g.	5.2 × 10^15^ v.g.	4.1 × 10^15^ v.g.	7.0 × 10^15^ v.g.	2.9 × 10^14^ v.g.
Anion-exchange column purification^c^	3.2 × 10^15^ v.g.	3.3 × 10^15^ v.g.	2.1 × 10^15^ v.g.	4.9 × 10^15^ v.g.	2.0 × 10^14^ v.g.
Final product^c^^,^^d^	2.9 × 10^15^ v.g.	2.7 × 10^15^ v.g.	2.0 × 10^15^ v.g.	3.9 × 10^15^ v.g.	1.8 × 10^14^ v.g.
Final product^e^	3.7 × 10^14^ v.g.	4.3 × 10^14^ v.g.	3.0 × 10^14^ v.g.	4.0 × 10^14^ v.g.	8.7 × 10^13^ v.g.
Recovery^f^	60%	63%	51%	70%	69%
Total recovery^g^	22%	27%	20%	46%	50%
Empty capsids (empty capsids/total particles)	3.8% (195/5,168)	5.2% (230/4,404)	2.6% (79/3,013)	7.3% (343/4,684)	3.8% (211/5,561)

Contaminating empty capsids in the final product were determined by EM.

a The standard plasmid used for qPCR was different from the one used for dsAAV measurement as described in Materials and Methods.

b The initial volume of medium was 1,120 mL.

c The titers were measured using ITR-targeted primers.

d The volume of final product was 13 mL.

e The titers were measured using non-ITR-targeted primers.

f The recovery was calculated by dividing the titer of “anion-exchange column purification” by the titer of “post-TFF.”

g Total recovery was calculated by dividing the titer of “final product measured using ITR-targeted primers” by the titer of “pre-TFF.”

### Qualification of the Final AAV9-dsEGFP Preparation

To assess reproducibility, AAV9-dsEGFP purification was replicated three times (Figure 2; Figure S3). The final products contained the three major highly pure protein bands that represent the AAV9 capsid proteins, VP1 (82 kDa), VP2 (67 kDa), and VP3 (60 kDa). The total capsid protein (VP1+VP2+VP3) pixel intensity was 94.9% ± 1.1% (n = 3), as determined by ImageJ densitometry of the SDS-PAGE gradient gels stained with Oriole fluorescent gel stain, and the capsid proteins derived from rAAV9 were determined by gels immunoblotted with the monoclonal antibody B1 (Figures 2D and 2E; Figures S3A, S3B, S3D, and S3E). Of the purified AAV9-dsEGFP particles, 96.1% ± 1.1% (n = 3) contained the viral genome, as determined by negative-stain EM (Figure 2F; Figures S3C and S3F). We also assessed the purified AAV9-dsEGFP with analytical ultracentrifugation (AUC) (Figure 2G). Unlike the EM result, it suggested that the final product contained approximately 26% of empty particles. The resultant total titer was 2.5 ± 0.4 × 10^15^ v.g., as measured using ITR-targeted primers (n = 3) (Table 1), or 3.7 ± 0.7 × 10^14^ v.g., as measured by EGFP-targeted primers. To assess the removal of human genomic DNA contamination likely derived from HEK293EB cells, we analyzed the final purified rAAV9 preparation by qPCR using human glyceraldehyde 3-phosphate dehydrogenase (*GAPDH*) primers (Table 2). No copy of human *GAPDH* (detection limit of one copy of human *GAPDH*) was detected in the final product (containing 1.0 × 10^9^ v.g.) and in the solvent of the final product; however, 2.6 copies of human *GAPDH* were detected in the post-tangential flow-filtration (post-TFF) solvent (containing 1.0 × 10^9^ v.g.). Therefore, human genomic DNA contamination can be removed with the chromatographic purification process described here. Furthermore, we evaluated the efficiency of the purified AAV9-dsEGFP to transduce HEK293EB cells (Table 3). The three purified replicates did not significantly differ in terms of transduction efficiency. Altogether, our protocol achieves reproducible purification of infectious AAV9-dsEGFP.

**Table 2:** Levels of Contaminating Human Genomic DNA Quantified in the Final Preparation of AAV9*-*dsEGFP

rAAV9 (1.0 × 10^9^ v.g.)	Human Genomic DNA Contamination	
Benzonase Treatment	Final Product^a^	Post-TFF
**+**^b^	<1 copy^c^	<1 copy^c^
**−**d	<1 copy^c^	2.6 copies

a Final products of three trials (AAV9-dsEGFP) were examined.

b Benzonase treatment (+) represents the contaminating human genomic DNA in rAAV9 particles.

c Less than one copy means below the detection limit.

d Benzonase treatment (−) represents the contaminating human genomic DNA in and outside of rAAV9 particles.

**Table 3:** The Proportion of EGFP-Positive HEK293EB Cells and Intensity of EGFP Fluorescence in Cells Transduced with Final rAAV9

Sample (v.g./cell)	5 × 10^6^		
Trial	1	2	3
EGFP-positive	11.3%	10.0%	10.4%

No significant difference was observed among the three preparations by one-way ANOVA; p = 0.343. The significance threshold was set at 0.05.

### Application Scope and Reliability

**Figure 3:**
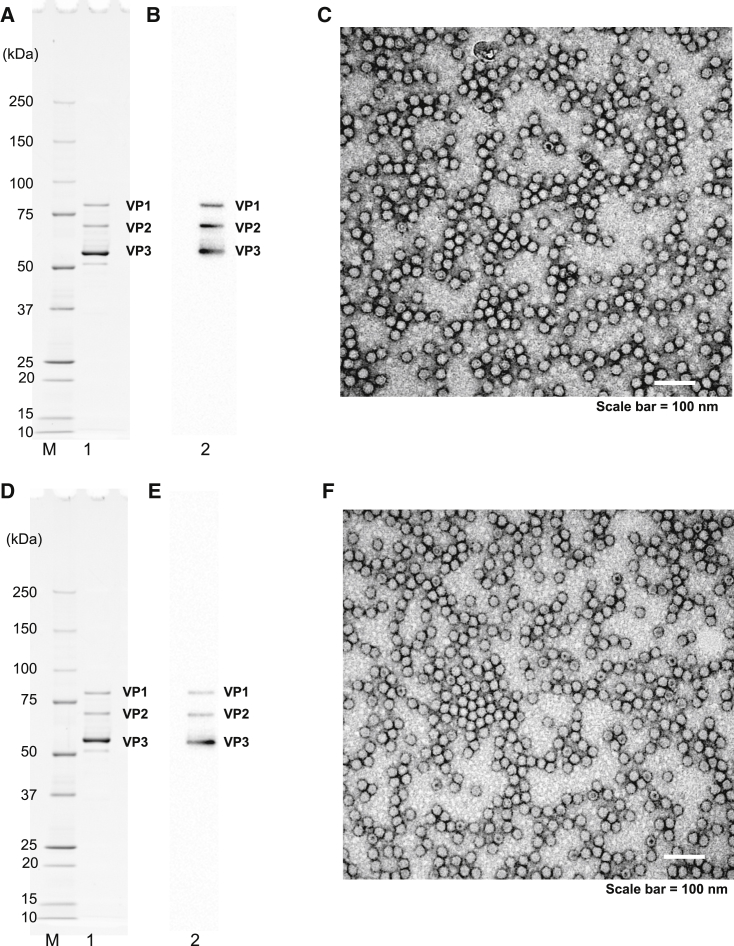
Laboratory-Scale Purification of AAV9-dsLuc or AAV9-ssLuc (A–F) Purity assessment of (A–C) AAV9-dsLuc and (D–F) AAV9-ssLuc. The preparation of final rAAV9 product was analyzed by (A and D) 5%–20% (v/v) gradient gel SDS-PAGE with Oriole fluorescent staining, (B and E) western blotting, and (C and F) EM (negative staining). Lane 1 and lane 2, final purified rAAV9 product. The three bands represent the AAV9 capsid proteins VP1 (82 kDa), VP2 (67 kDa), and VP3 (60 kDa).

**Figure 4:**
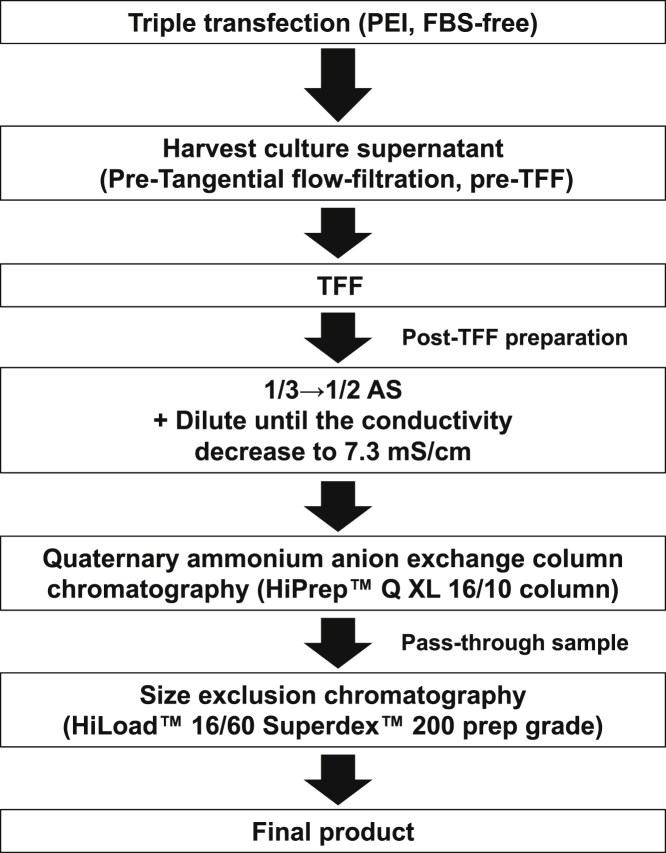
Schematic Representation of rAAV9 Production and Purification HEK293EB cells were transfected with three plasmids (*cis* AAV vector plasmid, *trans* plasmid, and helper plasmid) using polyethyleneimine (PEI, Polyethyleneimine Max) and maintained in DMEM without serum. After collecting the culture supernatant, it was ultrafiltrated by TFF with a hollow fiber using the KrosFlo Research Ili system. After reducing the amount of protein debris by 1/3-saturated AS precipitation, rAAV9 was precipitated in 1/2-saturated AS solution (1/3→1/2 AS). The precipitated rAAV fraction was loaded onto a quaternary ammonium anion-exchange column. The pass-through fraction was finally purified by size-exclusion chromatography.

To examine whether our purification protocol could be applied to different AAV constructs, we purified rAAV9 with dsAAV-CBA-red firefly luciferase (AAV9-dsLuc) and single-stranded AAV-CMV-RFLuc (AAV9-ssLuc) transgenes. The same purification procedure as used to purify AAV9-dsEGFP was performed. AAV9-dsLuc and AAV9-ssLuc preparations are shown in Figures 3A–3C and Figures 3D–3F, respectively. The final products contained the three major highly pure protein bands representing the AAV9 capsid proteins, as observed by SDS-PAGE and western blotting (Figures 3A, 3B, 3D, and 3E). The total capsid protein (VP1+VP2+VP3) intensity was 84% for AAV9-dsLuc and 86% for AAV9-ssLuc, as determined by densitometry using ImageJ. Of the purified rAAV9 particles, 92.7% (4,341 of 4,684 particles) of AAV9-dsLuc and 96.2% (5,350 of 5,561 particles) of AAV9-ssLuc contained the viral genomes (Figures 3C and 3F). The resultant titer of pure AAV9-dsLuc was 3.9 × 10^15^ v.g., as measured using ITR-targeted primers (4.0 × 10^14^ v.g., as measured by Luc-targeted primers), and that of pure AAV9-ssLuc was 1.8 × 10^14^ v.g., as measured using ITR-targeted primers (8.7 × 10^13^ v.g., as measured by Luc-targeted primers) (Table 1). Thus, we conclude that our purification procedure was also applicable for ssAAV and dsAAV of different transgenes. The procedures for rAAV9 production and purification used in our study are schematically shown in Figure 4. Three plasmids were transfected into HEK293EB cells using polyethyleneimine; cells were maintained in DMEM without serum. Five days later, the culture supernatant was collected and ultrafiltrated using TFF. The ultrafiltrated sample was precipitated by 1/3→1/2 AS treatment. Subsequently, the sample was purified by quaternary ammonium anion exchanger. The column resin retained the impurities and empty capsids; purification of the pass-through fraction containing rAAV9 was performed using size-exclusion chromatography.

## Discussion

We recently developed an efficient protocol for rAAV1 preparation using chromatographic purification and excluding ultracentrifugation.^11^ In this study, we attempted to optimize this procedure for purifying rAAV9 preparations. rAAV9 could be crudely purified by precipitation using saturated ammonium sulfate (1/3→1/2 AS), the same as rAAV1^11^ (Figure 1). During rAAV1 purification, rAAV1 and a 200-kDa impurity were bound to the anion-exchange column, but the 200-kDa impurity was separated from the final product by stepwise NaCl gradient elution. The 200-kDa impurity was also observed during the rAAV9 purification steps (white arrowhead in Figure 2A, lanes 1–5). In contrast to rAAV1 purification, this impurity bound to the anion- exchange column, whereas rAAV9 passed through the column; thus, this impurity was removed just by using the anion-exchange column (Figure 2A). Consistent with our results, Zhou et al.^25^ also reported that efficient binding of rAAV9 to either anion-exchange or cation-exchange resin was difficult. These results indicated that rAAV9 adsorption onto the anion exchanger markedly differed from that of rAAV1 during purification. A possible factor affecting rAAV adsorption onto the anion-exchange column may be the rAAV’s isoelectric point (pI). Venkatakrishnan et al.^26^ revealed that the unique N-terminal domain of VP1 undergoes a pH-induced, reversible change of folding that results in the loss or gain of its α-helical structure, does not disrupt capsid integrity, and is likely facilitated by changes in its pI. However, they also reported that no significant difference in pI was observed between AAV1 and AAV9. Moreover, both rAAV1 and rAAV9 were dissolved in a buffer at pH8.0 during our purification procedure. Thus, pI seems unlikely to be the critical determinant of the difference in rAAV1 and rAAV9 adsorption onto the anion exchanger. Small differences in amino acid side chains exposed on the rAAV surface may potentially contribute to its adsorption onto the anion-exchange column. Interestingly, AAV2.5, which was generated using the AAV2 capsid by substituting only five residues of AAV1, is distinct from AAV2 in terms of capsid characteristics.^27^ Although AAV1 shares 84.6% of capsid amino acid sequence identity with AAV9, a difference of several residues may affect the surface charge, leading to differences in rAAV1 and rAAV9 purification methods using ion-exchange chromatography.

rAAV9 purification has been reported previously.^17^, ^25^, ^28^ Lock et al.^17^ purified rAAV9 from culture supernatants using iodixanol gradient ultracentrifugation. The rAAV9 product was highly pure, and the genomic titer was comparable with our final rAAV9 preparation; however, an ∼4.7 times larger cell growth area than in our protocol was used to produce rAAV9. Zhou et al.^25^ reported that rAAV9 extracted from cell lysates did not bind efficiently onto the anion-exchange column under low-salt loading conditions (25 mM NaCl) at pH 8.5. Therefore, both ceramic hydroxyapatite chromatography and cation-exchange (Poros 50HS) chromatography were subsequently performed in the presence of polyethylene glycol. However, the final rAAV9 preparation was contaminated with ∼30% of empty capsids; thus, CsCl ultracentrifugation was used to remove the empty capsids for *in vivo* transduction experiments. Potter et al.^28^ purified rAAV9 from cell lysates by cation-exchange chromatography at a lower pH (pH 3.9), but their method also resulted in a highly contaminated final preparation containing empty capsids, according to their EM analysis. Other methods of affinity chromatography for rAAV9 purification have also been reported.^29^, ^30^ An affinity resin derivatized with single-domain monospecific antibody fragments against AAV9 capsids (Poros Capture Select AAV9) is available. Nass et al.^31^ demonstrated that the empty capsids and fully packaged virions were bound to the column under neutral pH conditions, and fully packaged rAAV9 was 11%, whereas the empty capsids were 88%, eluted by acidic conditions of pH 3.0–2.0. The acidic solution may be able to reduce the infectivity of rAAVs, and additional purification steps are required to separate the empty capsids from rAAV particles. Furthermore, this resin is too expensive to promote gene therapy by rAAV9 more widely ($2,735 for 25 mL). In contrast to previous reports, our successful purification is a significant methodological advancement. The first advantage of our protocol is the simplicity of the purification steps. Simple purification using anion-exchange and size-exclusion chromatography sufficiently remove most cellular contaminants and empty capsids from rAAV particles. This eliminates the laborious purification steps and multiple ion-exchange columns. Second, our protocol provides a final rAAV9 preparation of high quality and high yield despite its simplicity. The total titer of the final rAAV9 product in our protocol was 2.5 ± 0.4 × 10^15^ v.g., as measured using ITR-targeted primers (3.7 ± 0.7 × 10^14^ v.g., as measured using EGFP-targeted primers), which is comparable with or higher than that obtained using other protocols. In our final preparations, 96.1% ± 1.1% of the particles contained the viral genome, according to our negative-stain EM analysis (Figure 2F; Figures S3C and S3F). We also assessed the final AAV9-dsEGFP with AUC and observed about 38.8% of fully packaged virions and 25.7% of empty capsids in our final preparations (Figure 2G). Burnham et al.^32^ reported that fully packaged rAAV9 harboring a 2,050-nt genome were 34.4% ± 0.7% of the total in the final preparation (n = 5). Even though this is only a basic comparison of these values and our results, we believe that our simple purification method is reasonably similar. A method to evaluate the difference between EM and AUC and to identify the approximately 30% of intermediated particles (fragmented virus genome containing virions) is unclear at this moment. The full particles determined by EM may not be the veritable full particles when measured by AUC. Although EM has been conventionally used for evaluating the empty particle/full particle ratio, we may need to assess quality using both EM and AUC in the future. To achieve that, it is important to accumulate much data comparing EM and AUC moving forward. Third, our purification protocol can be used regardless of the type of AAV genome constructs (i.e., ss or ds, genome size, and GC content) (Figure 3; Table 1). Taken together, the procedures we describe here are fast, convenient, and versatile and efficiently remove empty capsids, producing high yields of highly pure rAAV9 for potential clinical use.

Contaminating empty capsids in the final rAAV preparations often compete for cell surface receptors and, consequently, prevent efficient transduction^33^ and can induce immune responses.^34^ To avoid this, empty particles must be cleared. We speculate that, in our purification steps, empty particles were initially removed by AS treatment. Although the starting material, a non-purified sample, contains many empty particles,^35^ the AS-treated sample contained fewer empty particles (Figure S2). We predict that the difference in surface structure between full particles and empty particles influences the hydrophobic interaction with AS; as a result, a certain level of empty particles was removed by AS treatment. Furthermore, we previously demonstrated efficient purification procedures to eliminate the contaminating empty capsids from rAAV1 and rAAV8 preparations.^10^, ^11^ We also demonstrated that the pI differed according to the presence or absence of the viral genome in AAV particles.^10^ Using similar strategies, many others have also developed procedures for efficiently separating the empty capsids from packaged rAAV particles based on pI differences using ion-exchange chromatography.^10^, ^11^, ^15^, ^36^, ^37^, ^38^ Qu et al.^36^ successfully removed AAV2 empty capsids using an cation-exchange resin, POROS 50HS, followed by an anion-exchange resin, Q-Sepharose^xl^. Using this method, they could obtain rAAV2 preparations that contained less than 20% of empty capsids of the total particles. Lock et al.^37^ separated empty capsids from rAAV8 particles by charge using a monolithic anion-exchange column, CIM-QA disk. They also successfully obtained rAAV8 particles containing 6.3% of empty capsids. In many procedures, rAAV particles were bound to ion exchangers first, and rAAV particles were separated from impurities or empty capsids by subsequent, stepwise gradient elution, similar to our rAAV1 purification.^11^ Contrary to other serotypes, rAAV9 particles are unlikely to bind efficiently to an anion exchanger under common conditions. Taking advantage of this rAAV9 characteristic, we successfully removed empty capsids from final rAAV9 products by passing them through the anion-exchange column. Although the mechanisms underlying this rAAV9 characteristic remain unknown, our results provide novel insights into establishing efficient purification strategies for removing the empty capsids derived from other natural or synthetic serotypes. In consideration of the AUC result, another chromatography step may be needed to further remove empty particles following the anion-exchange column to remove impurities and some empty particles.

Although our procedure can be successfully applied to laboratory-scale rAAV9 purification, it can also be applied to large-scale rAAV9 purification. For example, Grieger et al.^38^ reported that functional rAAVs could be obtained from suspended cells grown in animal component-free medium. In combination with their suspension and xeno-free culture system, our purification method will be easily scalable and adapted for current good manufacturing practice. In addition, our ultracentrifugation-free and CsCl-free procedure minimizes the risk of acute toxicity. Therefore, our streamlined, scalable, high-performance anion-exchange and size-exclusion chromatography protocols should facilitate future clinical studies using purified rAAV9.

## Materials and Methods

### Cell Culture

HEK293 or HEK293EB cells (HEK293 cells stably expressing the *E1* gene region and the *BCL-X*_*L*_ gene) were cultured at 37°C in a 5% CO_2_ atmosphere in DMEM (Sigma-Aldrich, St. Louis, MO) containing 8% (v/v) fetal bovine serum (FBS) (Biowest, Nuaillé, France) and penicillin and streptomycin (Sigma-Aldrich, St. Louis, MO). HEK293 cells were used for optimization of AS treatment conditions and in small-scale experiments. In laboratory-scale experiments, HEK293EB cells (∼3.2 × 10^9^ cells in total) were plated in 16 square culture dishes (245 × 245 × 18 mm; 500 cm^2^; Corning, New York, NY) with a surface area totaling 8,000 cm^2^. The initial volume of medium was 1,120 mL.

### Plasmids

The three *cis* AAV vector plasmids used were pdsAAV-CBA-EGFP, pdsAAV-CBA-RFLuc, and pssAAV-CMV-RFLuc. The pdsAAV-CBA-EGFP expresses the EGFP gene under the control of the CBA promoter with AAV type 2 ITR (donated by Dr. Arun Srivastava, University of Florida). In pdsAAV-CBA-RFLuc, the red firefly luciferase (RFLuc) fragment was amplified by PCR using the pCMV-RedFluc plasmid (Targeting Systems, El Cajon, CA) as a template and using the primers 5′-AACGAATTCGGATCCGCCACCATGGAAACAGAAAGAGAAGAAAACG-3′ and 5′-CTGGAATTCAAGCTTCTACCCACCTGCTTGAGGTTTCTTG-3′; the resultant PCR product was digested using NcoI and HindIII, and it was used to replace the EGFP in pdsAAV-CBA-EGFP. In pssAAV-CMV-RFLuc, the RFLuc fragment was amplified by PCR as described above and cloned into the EcoRI-HindIII site of pAAV-MCS (Agilent Technologies). The *trans* plasmid, pAAV2/9 (with the AAV type 2 *rep* gene and type 9 *cap* gene), was donated by James M. Wilson (University of Pennsylvania). The adenovirus helper plasmid, pHelper (with an essential region from the adenovirus genome), was purchased from Stratagene (La Jolla, CA).

### Production of rAAV9 and Preparation of the Crude rAAV9 Fraction

HEK293 or HEK293EB cells were grown for 2 days to reach 90% confluence. The *cis*, *trans*, and adenovirus helper plasmids were transfected at a ratio of 1:1:2^39^, ^40^ (44.8 μg of *cis* plasmid, 44.8 μg of *trans* plasmid, and 90.1 μg of pHelper per square dish) to cells maintained in DMEM without serum supplemented with GlutaMAX-І (Gibco, Life Technologies) and using polyethyleneimine (PEI Max, Polysciences, Warrington, PA) at a DNA:PEI ratio of 1:2.^17^ The culture supernatant was collected 120 h after transfection, filtrated through a 0.45-μm filter (Thermo Fisher Scientific, Waltham, MA), and ultrafiltrated by TFF using a hollow fiber cartridge (UFP-750-E-3MA; 750,000 nominal molecular weight cutoff; GE Healthcare, Westborough, MA) using the KrosFlo Research Ili TFF system (Spectrum Laboratories, Rancho Dominguez, CA). Subsequently, the crude rAAV9 fraction was treated with 25 U/mL benzonase (Novagen, San Diego, CA) for 30 min at 37°C. The reaction was terminated by adding 0.5 M ethylenediaminetetraacetic acid. The crude rAAV9 fraction was heated for 20 min at 50°C to denature the low-molecular-weight proteins. The denatured proteins were removed by three cycles of centrifugation at 13,100 × *g* for 10 min each. Next, 1/3 AS precipitation was performed by adding half of the sample volume of saturated AS to produce a final solution of 33% (w/v) AS, followed by centrifugation at 18,800 × *g* for 30 min at 4°C. rAAV9 was finally precipitated in 1/2 AS solution (by adding half of the original sample volume of saturated AS) (1/3→1/2 AS), followed by centrifugation at 18,800 × *g* for 30 min 4°C. The precipitated rAAV9 was dissolved in 20 mL of a buffer containing 3.3 mM morpholinoethanesulfonic acid (Dojindo, Kumamoto, Japan), 3.3 mM 4-(2-hydroxyethyl)-1-piperazineethanesulfonic acid (Dojindo), and 3.3 mM sodium acetate (Sigma-Aldrich, St. Louis, MO) (MHN buffer), pH 8.0, for laboratory-scale purification using a HiPrep Q XL 16/10 column (GE Healthcare, Uppsala, Sweden). The MHN buffer contained 50 mM NaCl and 0.01% (w/v) non-ionic surfactant Pluronic F-68 (Sigma-Aldrich).

### Purification of rAAV9 by Quaternary Ammonium Anion Exchangers and Size-Exclusion Chromatography

For laboratory-scale purification, 20 mL of 1/3→1/2 AS-treated sample was dissolved and diluted in MHN buffer until the conductivity of the solution decreased to 7.3 millisiemens/cm. The conductivity was measured using an electroconductivity sensor (LAQUAtwin, HORIBA, Tokyo, Japan). The diluted sample was loaded onto a quaternary ammonium anion exchanger with a bed volume of 20 mL (HiPrep Q XL 16/10) equilibrated with each buffered solution at a rate of 3 mL/min and controlled by a P1 peristaltic pump (Pump P1, GE Healthcare). The pass-through fraction was collected and ultrafiltrated using Ultracel 30 K centrifugal filters (Merck Millipore, Billerica, MA). We also collected the sample washed out through the anion-exchange column and the sample eluted from the anion-exchange column using MHN buffer (pH 8.0) containing 1 M NaCl and 0.01% (w/v) Pluronic F-68. The pass-through fraction was purified by size-exclusion (gel filtration) chromatography using a HiLoad 16/60 Superdex 200 preparation-grade column (GE Healthcare) and an ÄKTA Explorer 100 HPLC system (GE Healthcare) equipped with a 10-mL sample loop running the MHN buffer (pH 6.5) containing 300 mM NaCl and 0.01% (w/v) Pluronic F-68. Fractions were collected as follows: first segment, 0.2 column volume (CV), 5-mL fractions; second segment, 0.6 CV, 1-mL fractions; third segment, 0.3 CV, 5-mL fractions (1 CV = 120 mL). Peak fractions (fractions 15–27) were collected to obtain the final product. The purity of the final rAAV9 product was assessed by 5%–20% (v/v) gradient gel SDS-PAGE (SuperSep Ace, 5%–20%, 13-well, Wako, Osaka, Japan) and protein staining with either Quick Coomassie brilliant blue (Q-CBB, Wako, Osaka, Japan) or Oriole fluorescent gel stain (Bio-Rad, Hercules, CA) and by western blotting using an anti-AAV-capsid monoclonal antibody (B1, Progen, Heidelberg, Germany).

### qPCR

For purification of rAAV viral DNA, purified rAAV9 was treated with 125 U/mL benzonase and 2 mM MgCl_2_ for 30 min at 37°C. The viral genome DNA was extracted using the DNeasy Blood & Tissue Kit (QIAGEN, Hilden, Germany). The benzonase-resistant genome titer was measured by real-time qPCR (7500 Fast Real-Time PCR System, Applied Biosystems, Foster City, CA) using ITR-targeted primers (forward primer, 5ʹ-GGAACCCCTAGTGATGGAGTT-3ʹ; reverse primer, 5ʹ-CGGCCTCAGTGAGCGA-3ʹ),^41^ EGFP-targeted primers (forward primer, 5′-AGCAGCACGACTTCTTCAAGTCC-3′; reverse primer, 5′- TGTAGTTGTACTCCAGCTTGTGCC-3′), or RFLuc-targeted primers (forward primer, 5′- TCGACATCAGCTACCAGCAG-3′; reverse primer, 5′-ATCCCCAGACTGTGGTTCAG-3′) and SYBR green dye (SYBR Premix DimerEraser [Perfect Real Time] or TB Green Premix EX Taq II [Tli RNase H Plus], TaKaRa, Japan). The qPCR conditions were 10 s at 95°C, followed by 40 cycles at 95°C for 5 s and 60°C for 34 s. Linearized pdsAAV-CBA-EGFP DNA (digested by SacI restriction enzyme), pdsAAV-CBA-RFLuc DNA (digested by XmnI restriction enzyme), or pssAAV-CMV-RFLuc DNA (digested by BamHI restriction enzyme) were used as standard plasmids for rAAV9 with dsDNA or rAAV9 with ssDNA, respectively. The standard plasmids were purified with the QlAquick Gel Extraction Kit (QIAGEN).

### SDS-PAGE and Western Blot Analyses

The purified rAAV9 preparation was separated by 5%–20% (v/v) gradient polyacrylamide gel (Wako) in a running buffer containing SDS. To visualize and analyze the SDS-PAGE bands during AS treatment condition optimization and small-scale experiments, we used Q-CBB (Wako), and for the rest of the experiments we used Oriole fluorescent gel stain (Bio-Rad). The sensitivity of the Oriole gel stain is almost equal to that of silver staining.^42^ For western blotting, samples were transferred overnight onto polyvinylidene fluoride membranes (Merck Millipore). After transfer, membranes were blocked in 3% (w/v) BSA in Tris-buffered saline containing 0.05% (v/v) Tween 20 (TBST) and incubated with the primary antibody (1:1,000, monoclonal antibody B1, Progen, Heidelberg, Germany) for 1 hr at 4°C. After rinsing three times in TBST, the membranes were incubated with a horseradish peroxidase-labeled secondary anti-mouse antibody (1:10,000, GE Healthcare) for 1 hr at 4°C. Then the membranes were washed three times in TBST. The protein was detected using the enhanced chemiluminescence (ECL) Plus Western Blotting Detection System (GE Healthcare).

### Measurement of Contaminating Human Genomic DNA Contamination

Contaminating human genomic DNA in final preparations (AAV9-dsEGFP, three trials) was quantified by qPCR using human GAPDH-targeting primers (forward primer, 5ʹ-CTGGGCTACACTGAGCACC-3ʹ; reverse primer, 5ʹ-AAGTGGTCGTTGAGGGCAATG-3ʹ; NCBI: Pr032064752)^43^ and SYBR green dye (SYBR Premix DimerEraser [Perfect Real Time]). Human genomic DNA (Roche, Mannheim, Germany) was used as a standard DNA. For detection of contaminating human genomic DNA in rAAV9 particles, samples were treated with 125 U/mL benzonase and 2 mM MgCl_2,_ and then the viral DNA was purified with the DNeasy Blood & Tissue Kit. For detection of contaminating human genomic DNA outside of rAAV9 particles, the viral DNA was analyzed without benzonase treatment. The qPCR conditions were 10 s at 95°C, followed by 40 cycles of 95°C for 5 s and 60°C for 34 s.

### Measurement of the Transduction Efficiency of the Purified AAV9-dsEGFP

Triplicate wells containing 4.7 × 10^5^ HEK293EB cells were infected with purified AAV9-dsEGFP at 5.0 × 10^6^ v.g./cell. Three days after transduction, the proportion of EGFP-positive cells was measured by flow cytometry (FACSCantoII, Becton Dickinson, Franklin Lakes, NJ) with propidium iodide staining (Immunostep, Salamanca, Spain) to identify and exclude nonviable cells. Significant differences were determined using ANOVA by Microsoft Excel.

### Electron Microscopy

A carbon-stabilized copper grid (Nisshin EM, Tokyo, Japan) was placed onto a test sample and incubated for 1 min. The sample on the grid was then negatively stained for 1 min with 1% (w/v) uranyl acetate. Finally, the grid was examined using a H-7650 transmission electron microscope (Hitachi, Tokyo, Japan) at an accelerating voltage of 80 kV. The ratio of empty particles to encapsulated particles was determined by counting the number of packaged and unpackaged particles in the electron micrographs.

### AUC

Sedimentation velocity AUC analysis was conducted with a Proteome Lab XL-1 ultracentrifuge (Beckman Coulter, Indianapolis, IN). Four hundred microliters of AAV9-dsEGFP was loaded into the sample compartment of the centerpiece, and 400 μl of solvent was loaded into the reference compartment of the centerpiece. The four-hole rotor loaded the sample in the instrument, and it was equilibrated to a temperature of 20°C. Sedimentation velocity centrifugation was performed at 12,000 rpm and 20°C, and absorbance (260 nm) was used for analysis.

### AUC Data Analysis

The percentages of fully packaged virions, intermediate particles, and empty particles were determined by analyzing 92 scans and the Sedfit continuous-size C(S) distribution model. The following C(S) parameters were used for analysis: resolution, 100 S; S min, 20; S max, 120; frictional ration, 1.32859; *F* statistic, 0.68; buffer density, 1.0148; buffer viscosity, 0.0106. This model fits the data to the Lamm equation. The reliability of the analysis was confirmed by the goodness of fit (root-mean-square deviation [RMSD]).

## Author Contributions

T.T. designed the experiments, performed most of the experiments, analyzed data, and wrote the manuscript. H.O. and Y.M. conducted the experiments, analyzed data, and wrote the manuscript. K.A. performed some experiments. S.S., Y.K., H.C., and J.M. made experimental suggestions. A.I., M.O., and A.T. supervised the project. Y.H., T.S., and T.O. designed the experiments, conducted the experiments, analyzed data, and wrote the manuscript. All authors read and approved the manuscript.

## Conflicts of Interest

The authors have no conflicts of interest.

## Supplemental Information

Document S1. Figures S1–S3 and Supplemental Materials and Methods

Document S2. Article plus Supplemental Information

## References

[1] Nathwani A.C., Tuddenham E.G., Rangarajan S., Rosales C., McIntosh J., Linch D.C., Chowdary P., Riddell A., Pie A.J., Harrington C. (2011). Adenovirus-associated virus vector-mediated gene transfer in hemophilia B. N. Engl. J. Med..

[2] Muramatsu S., Fujimoto K., Kato S., Mizukami H., Asari S., Ikeguchi K., Kawakami T., Urabe M., Kume A., Sato T. (2010). A phase I study of aromatic L-amino acid decarboxylase gene therapy for Parkinson’s disease. Mol. Ther..

[3] Hauswirth W.W., Aleman T.S., Kaushal S., Cideciyan A.V., Schwartz S.B., Wang L., Conlon T.J., Boye S.L., Flotte T.R., Byrne B.J., Jacobson S.G. (2008). Treatment of leber congenital amaurosis due to RPE65 mutations by ocular subretinal injection of adeno-associated virus gene vector: short-term results of a phase I trial. Hum. Gene Ther..

[4] Miyoshi S., Tezuka T., Arimura S., Tomono T., Okada T., Yamanashi Y. (2017). *DOK7* gene therapy enhances motor activity and life span in ALS model mice. EMBO Mol. Med..

[5] Wirth T., Parker N., Ylä-Herttuala S. (2013). History of gene therapy. Gene.

[6] Gaudet D., Méthot J., Kastelein J. (2012). Gene therapy for lipoprotein lipase deficiency. Curr. Opin. Lipidol..

[7] Dias M.F., Joo K., Kemp J.A., Fialho S.L., da Silva Cunha A., Woo S.J., Kwon Y.J. (2018). Molecular genetics and emerging therapies for retinitis pigmentosa: Basic research and clinical perspectives. Prog. Retin. Eye Res..

[8] Hermens W.T., ter Brake O., Dijkhuizen P.A., Sonnemans M.A., Grimm D., Kleinschmidt J.A., Verhaagen J. (1999). Purification of recombinant adeno-associated virus by iodixanol gradient ultracentrifugation allows rapid and reproducible preparation of vector stocks for gene transfer in the nervous system. Hum. Gene Ther..

[9] Merten O.W., Gény-Fiamma C., Douar A.M. (2005). Current issues in adeno-associated viral vector production. Gene Ther..

[10] Okada T., Nonaka-Sarukawa M., Uchibori R., Kinoshita K., Hayashita-Kinoh H., Nitahara-Kasahara Y., Takeda S., Ozawa K. (2009). Scalable purification of adeno-associated virus serotype 1 (AAV1) and AAV8 vectors, using dual ion-exchange adsorptive membranes. Hum. Gene Ther..

[11] Tomono T., Hirai Y., Okada H., Adachi K., Ishii A., Shimada T., Onodera M., Tamaoka A., Okada T. (2016). Ultracentrifugation-free chromatography-mediated large-scale purification of recombinant adeno-associated virus serotype 1 (rAAV1). Mol. Ther. Methods Clin. Dev..

[12] Brument N., Morenweiser R., Blouin V., Toublanc E., Raimbaud I., Chérel Y., Folliot S., Gaden F., Boulanger P., Kroner-Lux G. (2002). A versatile and scalable two-step ion-exchange chromatography process for the purification of recombinant adeno-associated virus serotypes-2 and -5. Mol. Ther..

[13] Kaludov N., Handelman B., Chiorini J.A. (2002). Scalable purification of adeno-associated virus type 2, 4, or 5 using ion-exchange chromatography. Hum. Gene Ther..

[14] Zolotukhin S., Potter M., Zolotukhin I., Sakai Y., Loiler S., Fraites T.J., Chiodo V.A., Phillipsberg T., Muzyczka N., Hauswirth W.W. (2002). Production and purification of serotype 1, 2, and 5 recombinant adeno-associated viral vectors. Methods.

[15] Davidoff A.M., Ng C.Y., Sleep S., Gray J., Azam S., Zhao Y., McIntosh J.H., Karimipoor M., Nathwani A.C. (2004). Purification of recombinant adeno-associated virus type 8 vectors by ion exchange chromatography generates clinical grade vector stock. J. Virol. Methods.

[16] Tamayose K., Hirai Y., Shimada T. (1996). A new strategy for large-scale preparation of high-titer recombinant adeno-associated virus vectors by using packaging cell lines and sulfonated cellulose column chromatography. Hum. Gene Ther..

[17] Lock M., Alvira M., Vandenberghe L.H., Samanta A., Toelen J., Debyser Z., Wilson J.M. (2010). Rapid, simple, and versatile manufacturing of recombinant adeno-associated viral vectors at scale. Hum. Gene Ther..

[18] Vandenberghe L.H., Xiao R., Lock M., Lin J., Korn M., Wilson J.M. (2010). Efficient serotype-dependent release of functional vector into the culture medium during adeno-associated virus manufacturing. Hum. Gene Ther..

[19] Inagaki K., Fuess S., Storm T.A., Gibson G.A., Mctiernan C.F., Kay M.A., Nakai H. (2006). Robust systemic transduction with AAV9 vectors in mice: efficient global cardiac gene transfer superior to that of AAV8. Mol. Ther..

[20] Duque S., Joussemet B., Riviere C., Marais T., Dubreil L., Douar A.M., Fyfe J., Moullier P., Colle M.A., Barkats M. (2009). Intravenous administration of self-complementary AAV9 enables transgene delivery to adult motor neurons. Mol. Ther..

[21] Foust K.D., Nurre E., Montgomery C.L., Hernandez A., Chan C.M., Kaspar B.K. (2009). Intravascular AAV9 preferentially targets neonatal neurons and adult astrocytes. Nat. Biotechnol..

[22] Fu H., Dirosario J., Killedar S., Zaraspe K., McCarty D.M. (2011). Correction of neurological disease of mucopolysaccharidosis IIIB in adult mice by rAAV9 trans-blood-brain barrier gene delivery. Mol. Ther..

[23] Hayashita-Kinoh H., Yugeta N., Okada H., Nitahara-Kasahara Y., Chiyo T., Okada T., Takeda S. (2015). Intra-amniotic rAAV-mediated microdystrophin gene transfer improves canine X-linked muscular dystrophy and may induce immune tolerance. Mol. Ther..

[24] Okada T., Takeda S., Kinoh H. (2012). Drug delivery particle and method for producing the same. US patent.

[25] Zhou J., Yang X., Wright J.F., High K.A., Couto L., Qu G. (2011). PEG-modulated column chromatography for purification of recombinant adeno-associated virus serotype 9. J. Virol. Methods.

[26] Venkatakrishnan B., Yarbrough J., Domsic J., Bennett A., Bothner B., Kozyreva O.G., Samulski R.J., Muzyczka N., McKenna R., Agbandje-McKenna M. (2013). Structure and dynamics of adeno-associated virus serotype 1 VP1-unique N-terminal domain and its role in capsid trafficking. J. Virol..

[27] Bowles D.E., McPhee S.W., Li C., Gray S.J., Samulski J.J., Camp A.S., Li J., Wang B., Monahan P.E., Rabinowitz J.E. (2012). Phase 1 gene therapy for Duchenne muscular dystrophy using a translational optimized AAV vector. Mol. Ther..

[28] Potter M., Lins B., Mietzsch M., Heilbronn R., Van Vliet K., Chipman P., Agbandje-McKenna M., Cleaver B.D., Clément N., Byrne B.J., Zolotukhin S. (2014). A simplified purification protocol for recombinant adeno-associated virus vectors. Mol. Ther. Methods Clin. Dev..

[29] Wang Q., Lock M., Prongay A.J., Alvira M.R., Petkov B., Wilson J.M. (2015). Identification of an adeno-associated virus binding epitope for AVB sepharose affinity resin. Mol. Ther. Methods Clin. Dev..

[30] Terova O., Parra S., Clasen R., Hermans P. (2016). Innovative Downstream Purification Solutions for Viral Vectors: Enabling Platform Approaches to Advance Gene Therapies. Bioprocess Int..

[31] Nass S.A., Mattingly M.A., Woodcock D.A., Burnham B.L., Ardinger J.A., Osmond S.E., Frederick A.M., Scaria A., Cheng S.H., O’Riordan C.R. (2017). Universal Method for the Purification of Recombinant AAV Vectors of Differing Serotypes. Mol. Ther. Methods Clin. Dev..

[32] Burnham B., Nass S., Kong E., Mattingly M., Woodcock D., Song A., Wadsworth S., Cheng S.H., Scaria A., O’Riordan C.R. (2015). Analytical Ultracentrifugation as an Approach to Characterize Recombinant Adeno-Associated Viral Vectors. Hum. Gene Ther. Methods.

[33] Johnson J.S., Samulski R.J. (2009). Enhancement of adeno-associated virus infection by mobilizing capsids into and out of the nucleolus. J. Virol..

[34] Wang Z., Kuhr C.S., Allen J.M., Blankinship M., Gregorevic P., Chamberlain J.S., Tapscott S.J., Storb R. (2007). Sustained AAV-mediated dystrophin expression in a canine model of Duchenne muscular dystrophy with a brief course of immunosuppression. Mol. Ther..

[35] Sommer J.M., Smith P.H., Parthasarathy S., Isaacs J., Vijay S., Kieran J., Powell S.K., McClelland A., Wright J.F. (2003). Quantification of adeno-associated virus particles and empty capsids by optical density measurement. Mol. Ther..

[36] Qu G., Bahr-Davidson J., Prado J., Tai A., Cataniag F., McDonnell J., Zhou J., Hauck B., Luna J., Sommer J.M. (2007). Separation of adeno-associated virus type 2 empty particles from genome containing vectors by anion-exchange column chromatography. J. Virol. Methods.

[37] Lock M., Alvira M.R., Wilson J.M. (2012). Analysis of particle content of recombinant adeno-associated virus serotype 8 vectors by ion-exchange chromatography. Hum. Gene Ther. Methods.

[38] Grieger J.C., Soltys S.M., Samulski R.J. (2016). Production of Recombinant Adeno-associated Virus Vectors Using Suspension HEK293 Cells and Continuous Harvest of Vector From the Culture Media for GMP FIX and FLT1 Clinical Vector. Mol. Ther..

[39] Grimm D., Kern A., Rittner K., Kleinschmidt J.A. (1998). Novel tools for production and purification of recombinant adenoassociated virus vectors. Hum. Gene Ther..

[40] Salvetti A., Orève S., Chadeuf G., Favre D., Cherel Y., Champion-Arnaud P., David-Ameline J., Moullier P. (1998). Factors influencing recombinant adeno-associated virus production. Hum. Gene Ther..

[41] Aurnhammer C., Haase M., Muether N., Hausl M., Rauschhuber C., Huber I., Nitschko H., Busch U., Sing A., Ehrhardt A., Baiker A. (2012). Universal real-time PCR for the detection and quantification of adeno-associated virus serotype 2-derived inverted terminal repeat sequences. Hum. Gene Ther. Methods.

[42] Suzuki Y., Takagi N., Sano T., Chimuro T. (2013). Design and synthesis of a novel fluorescent protein probe for easy and rapid electrophoretic gel staining by using a commonly available UV-based fluorescent imaging system. Electrophoresis.

[43] Zhou H., Shi R., Wei M., Zheng W.L., Zhou J.Y., Ma W.L. (2013). The expression and clinical significance of HERC4 in breast cancer. Cancer Cell Int..

