# Supplementary material for: Highly Efficient Ultracentrifugation-free Chromatographic Purification of Recombinant AAV Serotype 9
Source: Mol Ther Methods Clin Dev. 2018 Nov 1;11:180–90. doi: 10.1016/j.omtm.2018.10.015 (PMC6276309; doi:10.1016/j.omtm.2018.10.015)
Supplement: Document S1. Figures S1–S3 and Supplemental Materials and Methods [file mmc1.pdf]

OMTM, Volume *11*

Supplemental Information

Highly Efficient Ultracentrifugation-free Chromatographic Purification of Recombinant AAV Serotype 9

Taro Tomono, Yukihiko Hirai, Hironori Okada, Yoshitaka Miyagawa, Kumi Adachi, Shuhei Sakamoto, Yasuhiro Kawano, Hideto Chono, Junichi Mineno, Akiko Ishii, Takashi Shimada, Masafumi Onodera, Akira Tamaoka, and Takashi Okada

Suppl 1

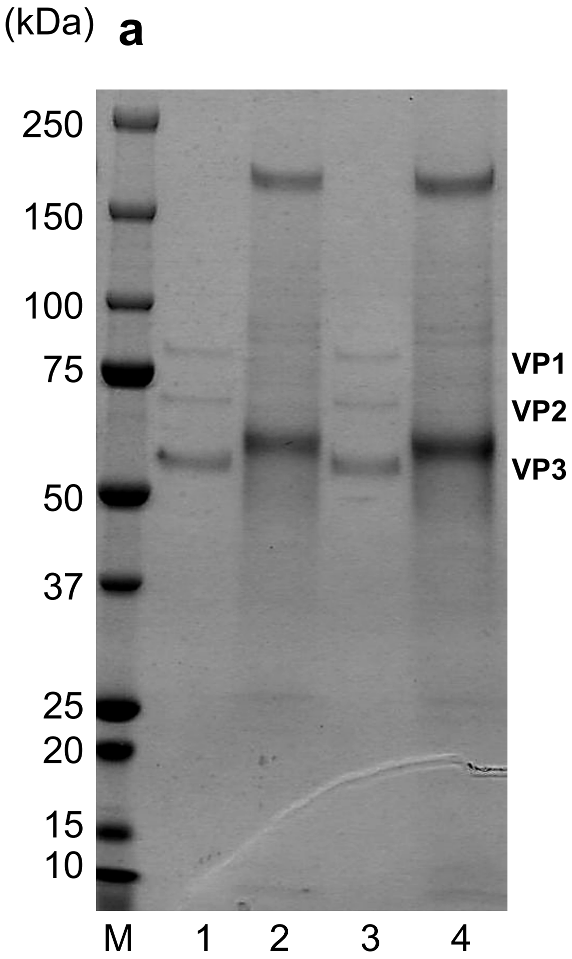

Suppl 1

b

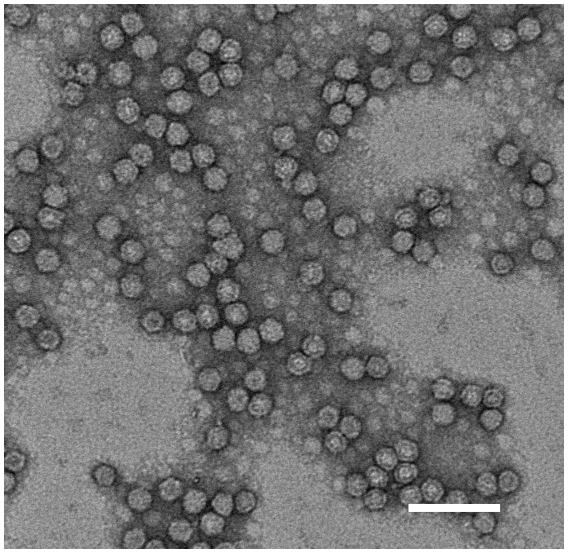

Scale bar=100 nm

d

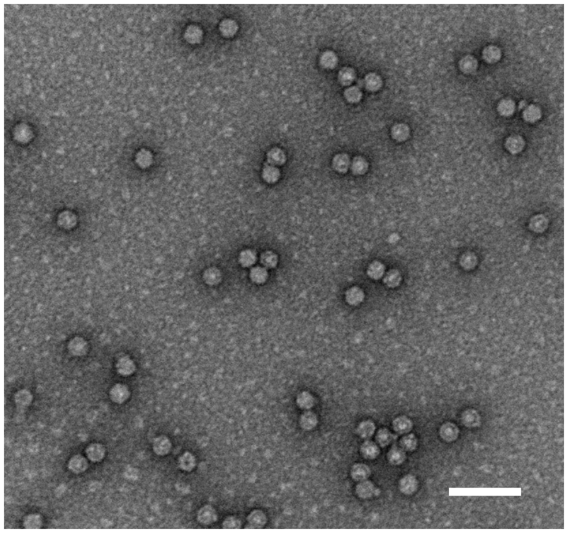

Scale bar=100 nm

c

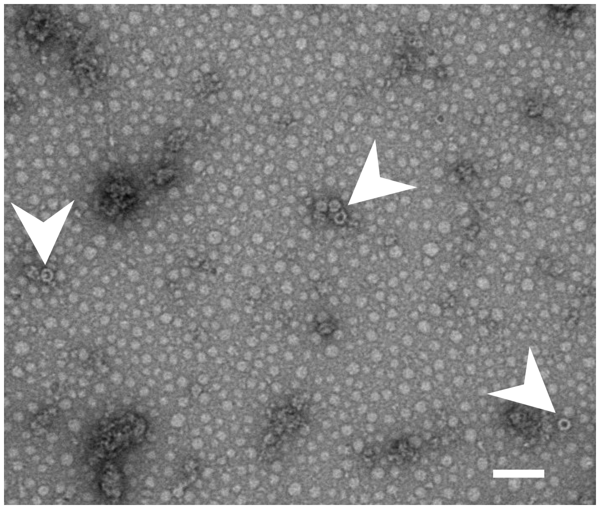

Scale bar=100 nm

e

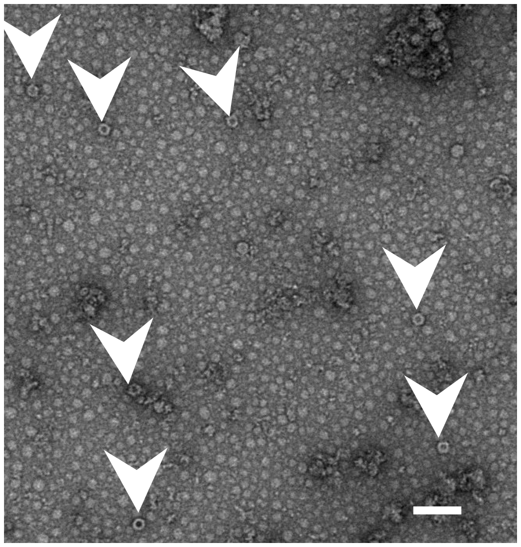

Scale bar=100 nm

Suppl 2

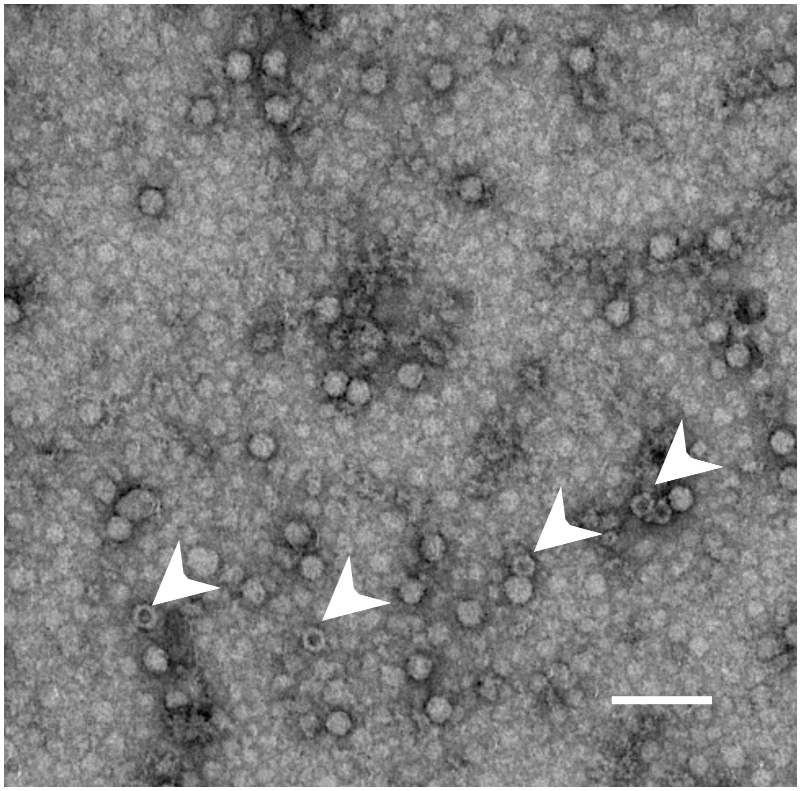

Scale bar = 100 nm

Suppl 3

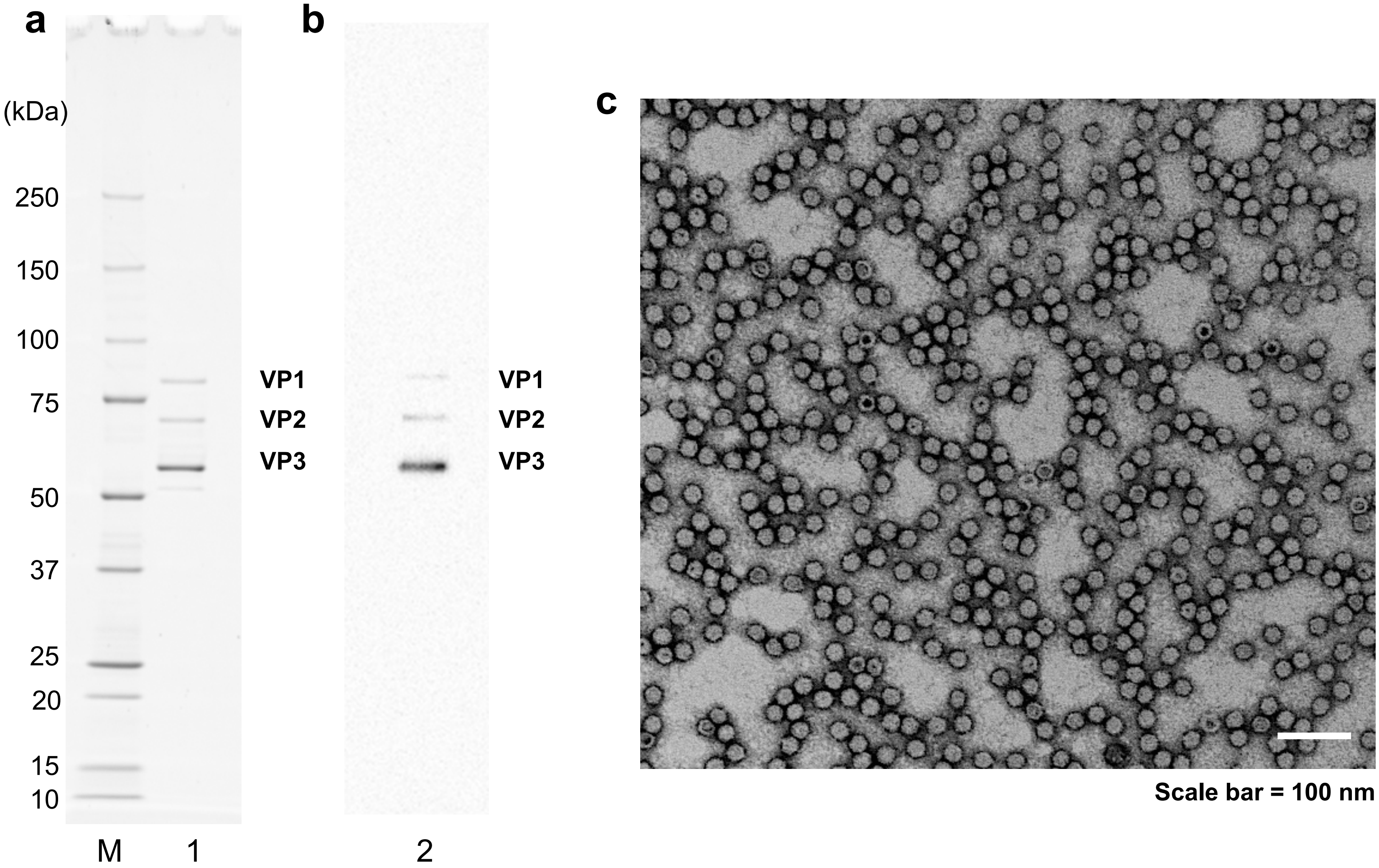
a
b
c
(kDa)
250
150
100
75
50
37
25
20
15
10
VP1
VP2
VP3
VP1
VP2
VP3
M
1
2
Scale bar = 100 nm

Suppl 3

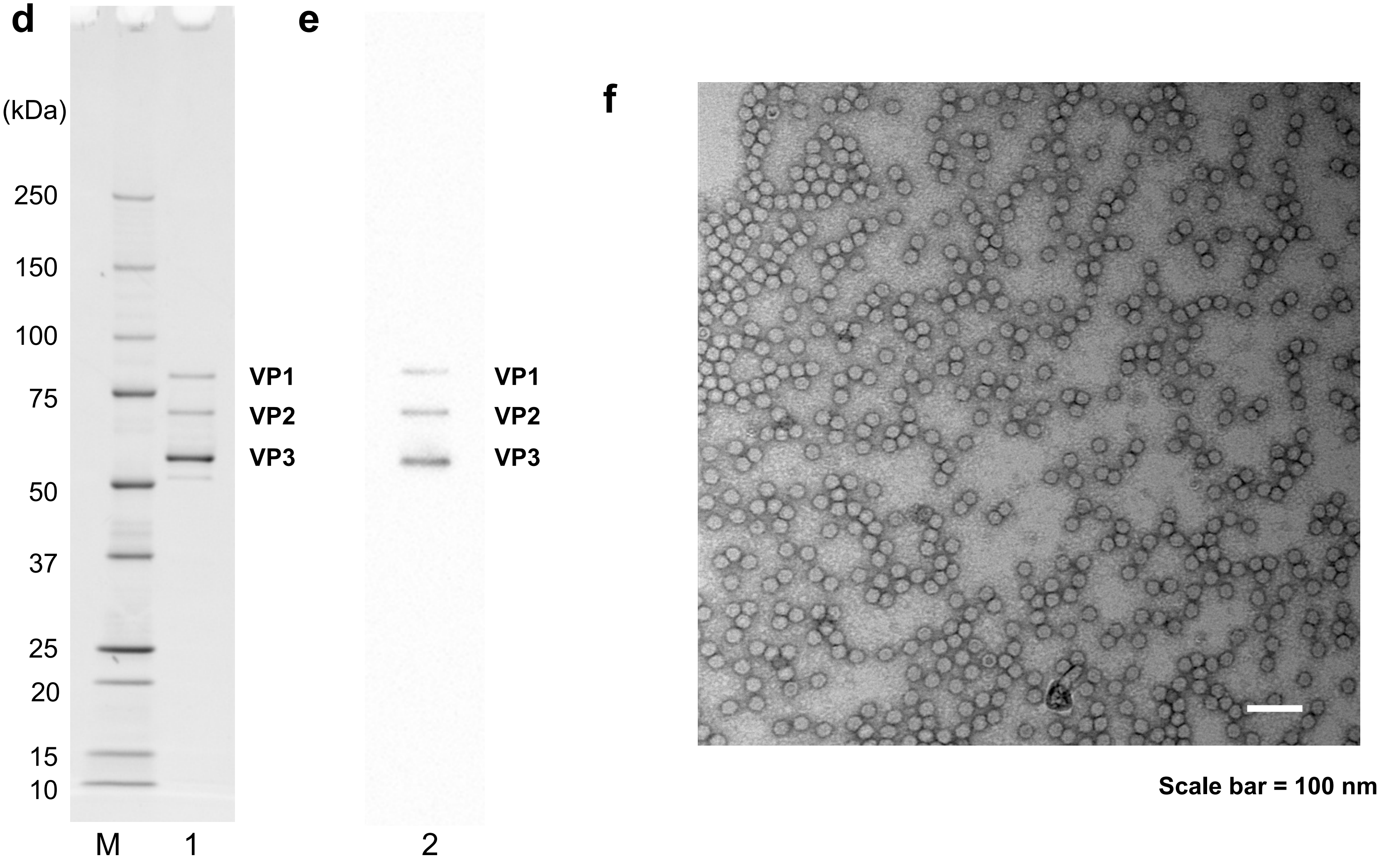

d
(kDa)
250
150
100
75
50
37
25
20
15
10
M
1
VP1
VP2
VP3
e
2
VP1
VP2
VP3
f
Scale bar = 100 nm

Supplementary Figure 1. Small-scale purification of AAV9-dsEGFP by quaternary ammonium anion exchange column chromatography

(a) The AAV9-dsEGFP preparation was analyzed by 5–20% (v/v) gradient gel SDS-PAGE with Q-CBB staining. The AS-precipitated rAAV9 was dissolved in 20 mL MHN buffer at pH 6.5 or pH 8.0 containing 50 mM NaCl and 0.01% (w/v) non-ionic surfactant Pluronic F-68. Subsequently, 1 mL of the sample was diluted 1:10 with the MHN buffer at each pH. The pass-through fraction was collected and ultrafiltered using Ultracel 30 K centrifugal filter units. We also collected the sample eluted from the HiTrap™ Q FF column with 3.3 mM MHN buffer (pH 8.0) containing 1 M NaCl and 0.01% (w/v) Pluronic F-68. M: protein size maker; Lane 1, pass-through fraction in pH 6.5 dilution buffer; Lane 2, column-bound fraction in pH 6.5 dilution buffer; Lane 3, pass-through fraction in pH 8.0 dilution buffer; Lane 4, column-bound fraction in pH 8.0 dilution buffer. The three bands represent the AAV9 capsid proteins VP1 (82 kDa), VP2 (67 kDa), and VP3 (60 kDa). Electron micrograph is shown for **(b)** the pass-through fraction and **(c)** the column-bound fraction in MHN buffer at pH 6.5. In the pass-through fraction, 90% of rAAV9 particles (359/399 particles) were fully packaged virions and almost all of the particles in the eluted fraction were empty (white arrowheads). Electron micrograph is also shown for **(d)** the pass-through fraction and **(e)** the column-bound fraction in the MHN buffer at pH 8.0. In the pass-through fraction, 93.7% of rAAV9 particles (623 / 665 particles) were fully packaged virions and almost all of the particles in the eluted fraction were empty (white arrowheads).

Supplementary Figure 2. Electron micrograph of diluted AS precipitated sample just before loading to anion-exchange column.

The diluted AS precipitated AAV9-dsEGFP preparation just before loading on an anion-exchange column (HiPrep™ Q XL 16/10 column) was analyzed using a negative-stain electron micrograph. The sample has a certain level of empty particles. White arrowheads show empty particles.

Supplementary Figure 3. Laboratory-scale purification of AAV9-dsEGFP for two additional trials

Purity assessment of AAV9-dsEGFP **(a-c)** for trial 2 and **(d-f)** for trial 3. The preparation of final rAAV9 product was analyzed by **(a, d)** 5–20% (v/v) gradient gel SDS-PAGE with Oriole fluorescent staining, **(b, e)** western blotting, and **(c, f)** electron microscopy (negative staining). M, protein size marker; Lane 1 and Lane 2, final purified preparation of rAAV9. The three bands represent the AAV9 capsid proteins VP1 (82 kDa), VP2 (67 kDa), and VP3 (60 kDa).

Supplementary methods (small scale)

Production of rAAV9

For small-scale production of rAAV9, ~2.4×10^9 HEK293 cells were plated in 12 square culture dishes (245 × 245 × 18 mm; 500 cm^2; Corning, New York, NY) with a total surface area of 6,000 cm^2 and 840 mL culture media. HEK293 cells were transfected with three plasmids, the *cis* AAV vector plasmid (pdsAAV-CBA-EGFP), the *trans* plasmid (pAAV2/9), and the adenovirus helper plasmid (pHelper) as described in Materials and Methods, "Production of rAAV9 and preparation of crude rAAV9 fraction".

Optimization of rAAV9 purification by quaternary ammonium anion exchange column chromatography

After reducing protein debris by precipitation using 33% (w/v) ammonium-sulfate (AS) added to the sample and centrifugation at 18,800 × *g* for 30 min at 4°C, rAAV9 was finally precipitated in 1/2 AS solution (by adding half the original sample volume of saturated AS) (1/3→1/2 AS) and centrifugation at 18,800 × *g* for 30 min at 4°C. Subsequently, the precipitated rAAV9 was dissolved in 20 mL MHN buffer (pH 8.0) containing 50 mM NaCl (Wako, Japan) and 0.01% (w/v) Pluronic F-68 (Sigma Aldrich). Then, 1 mL of the 1/3→1/2 AS-treated sample (corresponding to 1.2×10^8 HEK293 cells) was diluted 1:10 with MHN buffer at pH 6.5 or 8.0 containing 50 mM NaCl and 0.01% (w/v) Pluronic F-68 before loading onto a HiTrap™ Q FF (GE Healthcare, Uppsala, Sweden) column with a bed volume of 1 mL for small-scale preparation. The diluted sample was loaded onto the HiTrap™ Q FF column equilibrated with each dilution buffer at a rate of 1 mL/min using a peristaltic pump. Three protein bands were present in the pass-through fraction (**Supplementary Figure 1a**, Lane 1, pH6.5; Lane 3, pH8.0) and the column-bound sample contained protein impurities **(Supplementary Figure 1a;** Lane 2, pH6.5; Lane 4, pH8.0**)**. The pass-through fractions showed three clear bands by 5–20% (v/v) gradient gel SDS-PAGE with Q-CBB staining, suggesting that these fractions contained highly pure rAAV9. **Supplementary Figure 1b** shows an electron micrograph of the pass-through fraction in MHN buffer at pH 6.5. Of the purified rAAV9 particles, 90% (359/399 particles) were identified as packaged virions. **Supplementary Figure 1c** shows an electron micrograph of the column-bound fraction eluted in MHN buffer at pH 6.5. **Supplementary Figure 1d** and **Supplementary Figure 1e** show electron micrographs of the pass-through and column-bound fractions in MHN buffer at pH 8.0. Of the resultant purified rAAV9 particles, 93.7% (623/665 particles) were identified as packaged virions **(Supplementary Figure 1c)** and almost all of the empty capsids were bound to the HiTrap™ Q FF column **(Supplementary Figure 1e)**.
